# Programmed cell death-ligand 1 expression and immunoscore in stage II and III non-small cell lung cancer patients receiving adjuvant chemotherapy

**DOI:** 10.18632/oncotarget.18651

**Published:** 2017-06-27

**Authors:** Hidenobu Ishii, Koichi Azuma, Akihiko Kawahara, Norikazu Matsuo, Takaaki Tokito, Takashi Kinoshita, Kazuhiko Yamada, Tetsuro Sasada, Jun Akiba, Tomoaki Hoshino

**Affiliations:** ^1^ Division of Respirology, Neurology, and Rheumatology, Department of Internal Medicine, Kurume University School of Medicine, Kurume, Japan; ^2^ Department of Diagnostic Pathology, Kurume University Hospital, Kurume, Japan; ^3^ Cancer Vaccine Center, Kanagawa Cancer Center Research Institute, Yokohama, Japan

**Keywords:** non-small cell lung cancer, programmed cell death-ligand 1, immunoscore, tumor-infiltrating lymphocyte, adjuvant chemotherapy

## Abstract

Programmed cell death 1 (PD-1) receptor–ligand interaction is a major pathway that is often hijacked by tumors to suppress immune control. Immunoscore (IS), a combinational index of CD3 and CD8 tumor-infiltrating lymphocyte (TIL) density in the tumor’s center and invasive margin, is a new prognostic tool suggested to be superior to conventional tumor-staging methods in various tumors. This retrospective study aimed to investigate the prevalence and prognostic roles of PD-ligand 1 (PD-L1) expression and IS in non-small cell lung cancer (NSCLC) patients receiving adjuvant chemotherapy. PD-L1 expression and TIL density were evaluated by immunohistochemical analysis in 36 patients with stage II and III NSCLC. Tumors with staining in over 1% of their cells were scored as positive for PD-L1 expression, and we determined the median number of CD3- and CD8-positive TILs as the cutoff point for TIL density. To determine IS, each patient was given a binary score (0 for low and 1 for high) for CD3 and CD8 density in both the tumor center and invasive margin region. PD-L1 expression in tumor cells was observed in 61.1% (22/36) of patients. PD-L1 expression was significantly associated with high IS, and highest IS tended to have a favorable disease-free survival.

## INTRODUCTION

Lung cancer is the leading cause of death due to cancer worldwide [1]. Non-small cell lung cancer (NSCLC) accounts for 80% of all lung cancer cases, and the most effective treatment for NSCLC is surgical resection [2]. In addition, adjuvant chemotherapy improves survival in patients with completely resected stage II and III NSCLC [3–5]. However, approximately 50% of patients undergoing surgical resection relapse and die of recurrent disease within 5 years.

Immune checkpoint blockade with monoclonal antibodies has also recently emerged as a new therapeutic tool in oncology [6, 7]. Programmed cell death 1 (PD1), which belongs to the CD28 family of proteins, is a receptor expressed on the surface of T cells that regulates their activation and proliferation [6, 7]. Its ligand, programed cell death-ligand 1 (PD-L1), is frequently overexpressed in many types of human cancer [6]. Recent clinical trials have indicated that the inhibition of this pathway with anti-PD-1/PD-L1 antibodies exerts a promising antitumor effect against several human malignancies, including NSCLC, melanoma, and renal cell cancer [8–14]. Preliminary observations of patients with recurrent cancers have indicated that clinical responses to immune checkpoint blockers are associated with elevated tumor levels of immune inhibitory signals, such as PD-L1, CTLA-4 and with increased numbers of tumor-infiltrating lymphocytes (TILs) [6, 15–17].

Recently, several studies have demonstrated that the number, type, and location of TILs are important for predicting clinical outcome in various malignancies [18, 19]. Galon et al. reported that immunoscore (IS), evaluating the density of CD3 and CD8 TILs in the tumor center and invasive margin, was a new component for classifying cancer [20]. IS has been confirmed to be helpful in the prognosis and superior to the TNM classifications in early-stage colorectal cancer.

This retrospective study aimed to evaluate the prognostic role of PD-L1 expression and IS in stage II and III NSCLC patients who had received adjuvant chemotherapy and to investigate the association between PD-L1 expression and IS in them.

## RESULTS

### Patient characteristics

Among all screened patients, 36 were eligible for inclusion and were enrolled in this study. The clinical characteristics of these patients are shown in Table 1. All patients received platinum-based adjuvant chemotherapy after surgical resection.

**Table 1:** Patient and tumor characteristics

Number	36
Age	
Median	63
Range	43-75
Gender	
Male	21
Female	15
Smoking status	
Never	15
Former/Curent	21
Histology	
Non-squamous	28
Squamous	8
Satge	
IIA/IIB	12/6
IIIA/IIIB	16/2

### PD-L1 protein expression and IS

**Figure 1:**
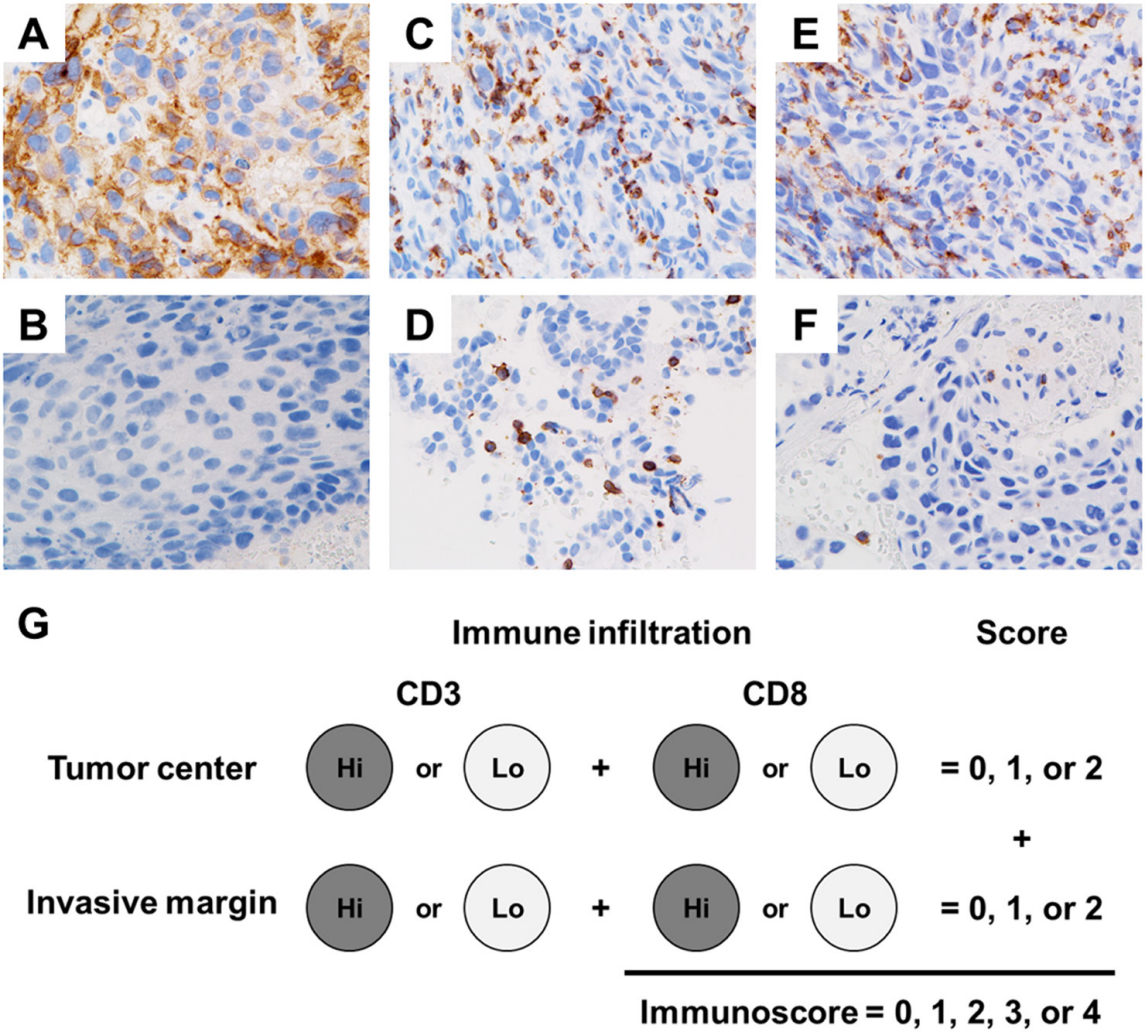
Representative programmed cell death-ligand 1 (PD-L1) and tumor-infiltrating lymphocytes (TIL) staining patterns in the tumor specimens Positive PD-L1 immunohistochemical staining pattern **(A)** and negative immunohistochemical staining pattern **(B)**. TILs were stained with CD3 (**C**, positive; **D**, negative) and CD8 (**E**, positive; **F**, negative) antibodies, and scoring was performed using a four-tier scale. Each tumor is categorized into Hi or Lo density for each marker in tumor region of tumor center and invasive margin. Patients were classified in five groups according to the IS score ranging from 0 to 4 **(G)**.

Immunostaining for PD-L1 was observed in the membrane and/or cytoplasm of the tumor cells and stromal lymphocytes. Representative PD-L1- and TIL-staining patterns in the tumor specimens are shown in Figure 1. Twenty-two (61.1%) patients exhibited positive PD-L1 staining in tumor (Table 2). The classification of the cohort according to IS is given in Figure 1G. Patients with a high degree of intratumoral immune cell infiltration had an IS of 3 to 4, whereas those with a low degree of immune cell infiltration had an IS of 0 to 2.

**Table 2:** Correlation of PD-L1 expression and TIL with patient characteristics

Factors	No	PD-L1 expression		*p*-value
		Positive	Negative	
Age				
<63	19	14	5	0.102
≥63	17	8	9	
Sex				
Male	21	12	9	0.563
Female	15	10	5	
Smoking				
Never	15	7	8	0.133
Former/Curent	21	15	6	
Histology				
Non-squamous	28	16	12	0.361
Squamous	8	6	2	
Pathological Stage				
IIA/IIB	18	13	5	0.172
IIIA/IIIB	18	9	9	
CD3 in tumor center				
High	18	14	4	0.040
Low	18	8	10	
CD3 in invasive margin				
High	18	12	6	0.494
Low	18	10	8	
CD8 in tumor center				
High	18	14	4	0.040
Low	18	8	10	
CD8 in invasive margin				
High	18	14	4	0.040
Low	18	8	10	
Immunoscore				
High (3-4)	15	12	3	0.049
Low (0-2)	21	10	11	

PD-L1, programed cell death-ligand 1; TIL, tumor infiltrating lymphocyte.

### Correlation of PD-L1 expression and TIL with patient characteristics

The correlation of PD-L1 protein expression and TIL density with NSCLC patients’ clinicopathological features is shown in Table 2. PD-L1 expression and CD8 TIL density in the tumor center and invasive margin were significantly correlated. Furthermore, PD-L1 expression was significantly associated with high IS scores. PD-L1 expression was not correlated with the other patient characteristics, such as age, gender, smoking status, histology (squamous cell carcinoma or non-squamous cell carcinoma), mediastinal lymph node metastasis (N0 or N1-2), or pathological stage (stage II or III).

### Survival analysis

**Figure 2:**
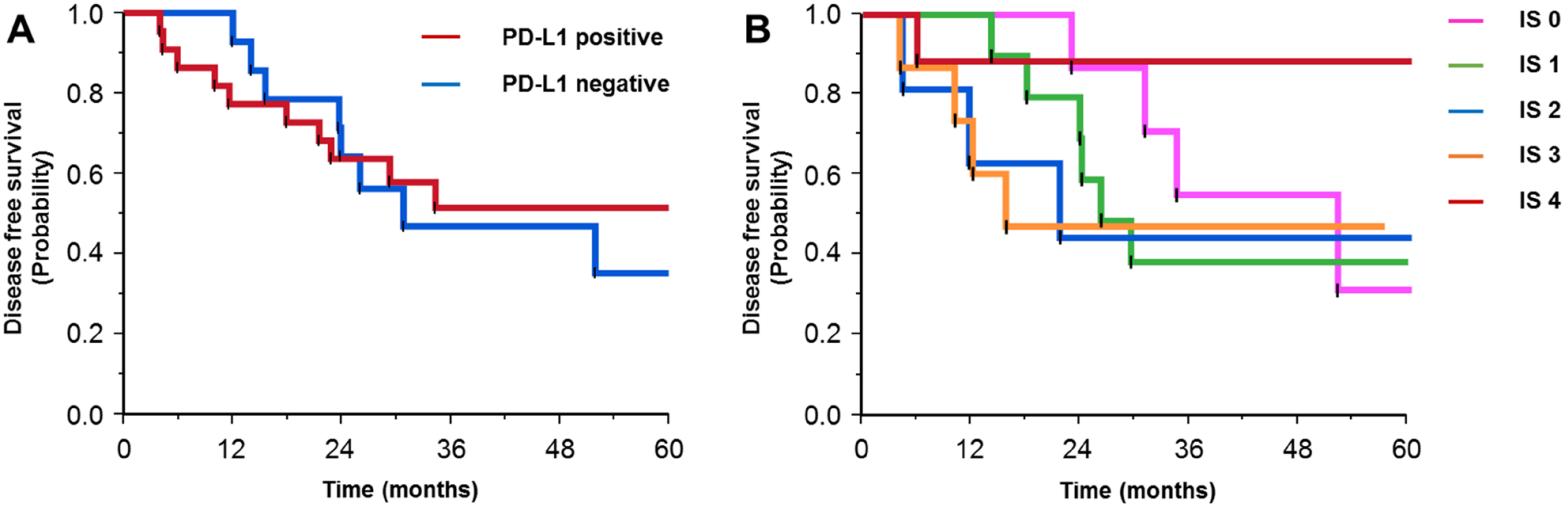
Kaplan-Meier curves for disease-free survival in patients with positive or negative expression of programmed cell death-ligand 1 **(A)**, and according to the immunoscore **(B)**.

During analysis, the median follow-up duration was 36.1 (range, 8.4–126.4) months. The correlation between patient characteristics and survival is shown in Table 3. PD-L1 expression was not correlated with disease-free survival (Figure 2A, DFS; median DFS in the PD-L1-negative group was 30.8 months, but that in the PD-L1-positive group was not reached, *p* = 0.776) and overall survival (OS; median OS in both groups was not reached, *p* = 0.836). Disease recurrence was present less frequently in patients with high stromal CD8 TIL density than in those with low CD8 TIL density (27.8%; 5 of 18 versus 72.2%; 13 of 18, *p* = 0.018). Among the five IS-classified cohorts, the cohort of score 4 had the longest DFS according to the Kaplan–Meier curves (Figure 2B).

**Table 3:** Analysis of prognostic factors for DFS and OS

Factors	No	mDFS(mo)	*p*-value	mOS(mo)	*p*-value
Age					
<63	19	51.9	0.407	NR	0.501
≥63	17	30.8		75.4	
Sex					
Male	21	29.3	0.725	NR	0.536
Female	15	NR		NR	
Smoking					
Never	15	30.8	0.158	NR	0.24
Former/Current	21	NR		NR	
Histology					
Non-squamous	28	29.3	0.049	NR	0.441
Squamous	8	NR		*NR*	
Stage					
IIA/IIB	18	NR	0.255	NR	0.322
IIIA/IIB	18	29.3		NR	
PD-L1 expression					
positive	22	NR	0.776	NR	0.836
negative	14	30.8		NR	
Immunoscore					
0	7	51.9		NR	
1	9	26.0		NR	
2	5	21.5		75.4	
3	7	15.6		39.6	
4	8	NR		NR	

mDFS, median disease-free survival; mOS, median overall survival; mo, month.

## DISCUSSION

The objective of adjuvant chemotherapy is to prevent relapse in patients having undergone complete surgical resection. Patients who may receive benefit from adjuvant chemotherapy should be selected more effectively, and to improve the prognosis, new strategies should be developed. Although immune checkpoint blockade with monoclonal antibodies has recently emerged as a new therapeutic strategy in several malignancies, the role of PD-L1 expression and TIL density in NSCLC patients receiving adjuvant chemotherapy remains largely unknown. Here, using immunohistochemistry, we examined PD-L1 expression and IS in NSCLC patients. We found a positive correlation between PD-L1 expression and IS, and high IS tended to be associated with favorable DFS.

IS is currently used as a prognostic tool for quantifying *in situ* immune cell infiltrates by evaluating the CD3- and CD8-positive TILs [21]. Previous studies have reported an association between the density of various types of TILs and prognosis in NSCLC [22]. Donnem et al. reported that high stromal CD8 T-cell density can predict favorable outcomes in patients with resected NSCLC [23]. Furthermore, several studies have reported that a strong immune component is predictive of a favorable response to chemotherapy in some types of cancer, including NSCLC [24–26]. Consistent with those studies, the present study also demonstrated that a higher stromal CD8 TIL density is significantly associated with the absence of recurrence and longer DFS. CD8, which is predominantly expressed on cytotoxic T cells, is a crucial component of the cellular immune system and pivotal to cell-mediated antitumor immune responses. These results indicate that CD8-positive T cells with cytotoxic activity play an important role in antitumor immunity and can circumvent many barriers inherent in the cancer-induced stroma through optimized specificity, activation, homing, and antitumor function [27].

Although previous studies have demonstrated that PD-L1 expression is associated with a poor clinical outcome in several malignancies [28–32], some findings remain controversial [33–36]. In our study, PD-L1 expression was not correlated with DFS or OS in patients with stage II and III NSCLC receiving adjuvant chemotherapy, consistent with the results in some previous studies [9–11]. Preliminary analyses in the phase III clinical trials of anti-PD-1/PD-L1 therapy suggested that PD-L1 expression in the tumor predicts response to these therapies. The significance of PD-L1 expression in adjuvant chemotherapy with PD-1/PD-L1 blockade therapy is currently unclear.

Our study had several limitations. A major weakness is that the number of patients studied was relatively small. Secondly, the information was collected retrospectively, and thirdly, the determination of PD-L1 expression in tumor samples was generally performed by immunohistochemistry using various antibodies. Further studies are warranted to harmonize and standardize testing for PD-L1 expression by immunohistochemistry in larger samples. Although randomized trials are needed to evaluate whether IS and PD-L1 expression predict the efficacy of adjuvant chemotherapy, it might be difficult to design a trial including study arm of patients without adjuvant chemotherapy, because adjuvant chemotherapy has been already established as a standard treatment option in patients with completely resected stage II and III NSCLC.

In conclusion, we have demonstrated a significant association between PD-L1 expression and IS: a highest IS provides long DFS in patients with stage II or III NSCLC receiving adjuvant chemotherapy. Further studies are warranted to clarify the role of PD-L1 expression and IS as well as the therapeutic effect of PD-1/PD-L1 blockade in clinical trials with these patients. Our findings may have important implications for PD-1/PD-L1 blockade therapy in patients with stage II and III NSCLC receiving adjuvant chemotherapy.

## MATERIALS AND METHODS

### Patients

We retrospectively screened consecutive patients who had been diagnosed with lung cancer and had undergone complete surgical resection at Kurume University Hospital between 2005 and 2014. Patients who had been diagnosed pathologically as having NSCLC, who had received adjuvant combined platinum-containing chemotherapy after surgical resection, and for whom adequate histological specimens containing abundant tumor cells were available, were eligible for inclusion. The present study was conducted in accordance with the provisions of the Declaration of Helsinki and was approved by the Institutional Review Board of Kurume University Hospital.

### Immunohistochemical analysis of PD-L1 proteins and CD8 T-cell infiltration

We used 4-μm-thick sections of formalin-fixated, paraffin-embedded (FFPE) tissue obtained from surgical resection. The sections were mounted on glass slides and then incubated with anti-rabbit monoclonal antibody against PD-L1 (abcam, Cambridge, UK) for immunohistochemical analysis using BenchMark XT (Ventana Automated Systems, Inc., Tucson, AZ, USA). Briefly, each slide was heat-treated using Ventana’s CC1 retrieval solution for 30 min and incubated with the PD-L1 antibody for 30 min. This automated system used the ultraVIEW DAB detection kit with 3, 3′-diaminobenzidine (DAB) as the chromogen (Ventana Automated Systems). Cases in which >1% of the tumor stained for PD-L1 were considered positive.

### Immunohistochemical analysis of TILs

After the tumor center and invasive margin area were reviewed, pathologic slides from FFPE tissues were stained with monoclonal antibodies to CD3 and CD8. Immunostaining for CD3 and CD8 (Leica Microsystems, Newcastle-upon-Tyne, UK) was performed on the same completely automated Bond-III system (Leica Microsystems) using onboard heat-induced antigen retrieval with epitope retrieval solution 2 for 10 min at 99°C, followed by incubation with the antibody for 30 min at room temperature. This automated system used a Refine polymer detection kit with horseradish peroxidase polymer as a secondary antibody and DAB, and incubation with secondary antibody was performed for 30 min at room temperature.

### Determination of IS

All immunohistochemical analyses were evaluated by two experienced observers who were unaware of the patients’ conditions. Spots on which the pathologists disagreed regarding the staining category were reviewed jointly by them, and a single consensus category was established. In addition, TILs were considered as immunostained CD3 and CD8 preparations, and scoring was performed using a four-tier scale. We determined the median number of CD3- and CD8-positive TILs as the cutoff point for high or low TIL density. Based on the established cutoff point, each patient was given a binary score (0 for low and 1 for high) for each immune cell type (CD3 and CD8) in each tumor region (tumor center and invasive margin). Patients were classified in five groups according to the IS score ranging from 0 to 4 (Figure 1G).

### Statistical analyses

Correlations between PD-L1 expression, TIL density, IS, and patient characteristics were analyzed using the chi-squared or Fisher’s exact test for categorical variables. We evaluated whether parameters, including PD-L1 expression and IS, were associated with the presence of recurrence, DFS, and OS of stage II and III NSCLC patients receiving adjuvant chemotherapy. DFS was defined as the period from surgery to recurrence or the last follow-up if no recurrence was observed. OS was measured from the administration of treatment or initial diagnosis until the date of death or last follow-up. The Kaplan–Meier method was used to assess the patients’ survival curves, and the log-rank test was used to evaluate the significance of differences between two groups. Multivariate regression was performed using the Cox proportional hazards model. All variables that had p values of <0.05 were included in the Cox model. All tests were two-sided, and differences were considered statistically significant at *p* < 0.05. All statistical analyses were conducted using JMP version 10 (SAS Institute Inc., Cary, NC).
